# Characterization of liquid products obtained from catalytic binary co-cracking of residual fuel oil with various waste plastics

**DOI:** 10.1038/s41598-022-15371-8

**Published:** 2022-06-29

**Authors:** Pamreishang Kasar, Md. Ahmaruzzaman

**Affiliations:** Department of Chemistry, National Institute of Technology, Silchar, 788 010 India

**Keywords:** Environmental sciences, Energy science and technology

## Abstract

Recycling polymeric waste and heavy oil residues are important for energy recovery and raw material processing. Catalytic pyrolysis is a unique technology used to generate alternative energy, and it can stands out to be one of the environmentally friendly and alternative routes for the generation of renewable energy. Limited study has been reported in the literature on the co-cracking of residual fuels with waste plastics to establish its properties and potential. In this study, we have characterized the products in liquid form resulting from the co-cracking of residual fuel oil (RFO) with plastic waste in an isothermal condition. The characterization was carried out using nuclear magnetic resonance (^1^H NMR & ^13^C NMR), Fourier transforms infrared spectroscopy (FTIR), gel permeation chromatography (GPC), bomb calorimetry, and ultimate analyzer, in addition to the characterization of the flashpoint, pour point, and density. As a result of co-cracking, the liquid exhibits a significant decline in the overall molecular weight and an increase in the content of saturated aliphatic carbon and a decrease in the protonated aromatic carbons with aliphatic compounds as the primary constituent were observed from the spectra, having a pour point of 291.15–192.15 K and high calorific values between 42–45 MJ/kg. The characteristics of the liquid reveal a synergistic effect of co-cracking and demonstrate the potential of the co-cracking process of waste plastics with residual fuel to be an alternate source of energy and added-value chemical product recovery routes.

## Introduction

The catalytic co-cracking of petroleum residual fuel oil (RFO)^1^ with various waste plastics was carried out to examine the process and products, which may have interesting patterns and may further open up more prospects to explore its possible enhancement for better applications. To understand the chemical transformation and chemical reactions which could have taken place in the process of co-cracking, it is not only important but necessary to characterize the resulting products. Consequently, the product classification will help determine the end use of the final products and will further facilitate any improvement or amendment in the reactor and variable processes under which cracking was undertaken. Investigation of the co-pyrolysis of blend waste of various plastics has revealed that the reaction process affects the characteristics of the individual plastics^2^. Further, simulation pyrolysis methods for single plastics^3–6^ have been adopted to go forward with the examination of the execution of the pyrolysis technique. Computer-aided simulation was also used for both simulating and performance analysis of waste lubricant oil through pyrolysis^3^. Most recently, the co-pyrolysis of plastic waste with waste lubricant oil was undertaken to produce a diesel-like product^7^. Co-pyrolysis of polymeric waste with oil shale shows an active course of decomposition with a positive synergistic effect of processing together in equal proportion and increase in the liquid yield, implying a promising way for the material to enter into waste management market^8^. Co-processing of vacuum residue with waste plastics shows a synergistic effect on activation energy^9^, the co-cracking of of waste motor oil with waste polyolefins increases the liquid yields and also improved the properties of liquid products, showing a synergistic effect of co-pyrolysis^10^. Co-pyrolysis of Brazilian crude oil with polypropylene (PP) at the temperature range of 673.15–73.15 K yielded 80% pyrolytic oil^11^. The thermal cracking of petroleum residue has resulted in liquid products of hydrocarbon as the primary product, while both gas and coke were formed during the process. Characterization of the liquid products, like the average structural parameters (ASP), their constituents, and other characteristics, will help understand the physic-chemical features. Various analysis methods, such as GPS, were employed to examine and comprehend the molecular weight distribution (MWD) of the polymers^12^. The degradation process and pyrolysis behavior of oil shale kerogen were executed using Thermogravimetric Analysis- Fourier-transform infrared spectroscopy (TGA-FTIR)^13^, while the FT-IR analysis technique was used to detect the functional groups in the inorganic and organic compound^14^. ^1^H and ^13^C NMR analysis methods were used to evaluate structural parameters of both high and low boiling petroleum cuts^15–17^.

The co-cracking of waste plastics with petroleum Residual Fuel Oil(RFO) may synergize the process of decomposition due to several electrophilic reactions and thereby resulting in the production of stabilized products. The molecular weight distribution products resulting from the cracking of plastics were also ascertained using size exclusion chromatography^18^ and reported that the molecular aromatic compounds in the liquid increase with the increase in the co-cracking temperature, thereby affecting the molecular weight. The chemical and physical properties of the diesel and gasoline fuels resulting from the thermal cracking of waste plastics were investigated and characterized^19^, while the characterization of petroleum residues was carried out using NMR and FT-IR analysis techniques^20^. Further, the characterization of liquid products resulting from the cracking of polyethylene was carried out by Horvat and Ng^21^.

Co-processing of low valued residues of the refineries (RFO) with specific waste plastics like PPI, PPX, HDPE, and BL to explore the possibilities of upgrading the low-value materials and to lighter fuel oil has not been reported earlier. The heavy oil residues, including RFO, which are less desired in the market, are piling up in the refineries as a result of intensifying efforts to meet the rising demand for the lighter fuel oil. Similarly, waste plastics have entered everywhere in our environment, including landfills, waterways, oceans, etc., thereby impacting the lives of its inhabitants.

Therefore, it is an indispensable choice to opt for these materials and dwell on these materials to convert them into value-added products by adopting an environmentally friendly method. Exploring alternate potential routes of adding value to the products in order to facilitate better and proper applications of the end products can play a significant role. Pyrolysis is one such method that can be adopted to co-process the two low value-added materials (Waste plastics and RFO) in an environmentally friendly approach to recover these wastes or upgrade them, thereby avoiding the pollution caused by them while simultaneously creating an alternative energy source.

Further, to understand the reactions which could have occurred during the co-cracking process, the characterization of the liquid products, in addition to the solid product^22^, was necessary. The main objective of the study was to understand the average structural parameters, molecular weight distribution, calorific values, flash point, pour point, and density, besides the elemental content in the liquid products. The information resulting from this analysis may facilitate further characterization and understanding of the products; thereby, the best potential result in terms of its applicability may be discovered for further application or investigation.

The novelty of this study is that the liquid products resulting from the co-cracking of the process have a high potential to be the sources of raw materials for other products. The suitability and the best potential of the products can be discovered by characterization of the products. Some of the primary analyses adopted in the study can pave the way for further study for optimization of the co-cracking process potential and applicability of the final products. This study is more significant and necessitated by the fact that the raw material used in the study are both considered either waste or low value material. Further, the plastics waste in particular is one among the major factor responsible for the environmental pollution and there is a paramount obligation for a proper management or alternative means of utilizing or conversion into lighter and higher value products. Moreover, the heavy oil are piling up in the refineries; and refieneries are looking for better means and process for its upgradation to recover more lighter fuel from it. Through this study, we are probing the characteristics of the liquid products resulted from the unique route of co-processing the two low valued material for possible conversion to higher value product while at the same time helping in the better waste management can be considered as legitimate effort.

## Experimental

### Materials and methods

The source of material and characteristics of RFO and PPX, including the process of co-pyrolysis, have been reported in the previous paper^1^. While the rest of the plastics waste materials (PPI, HDPE, and BL) were industrial waste collected from the industries in Guwahati.

### Pyrolyis

**Figure 1:**
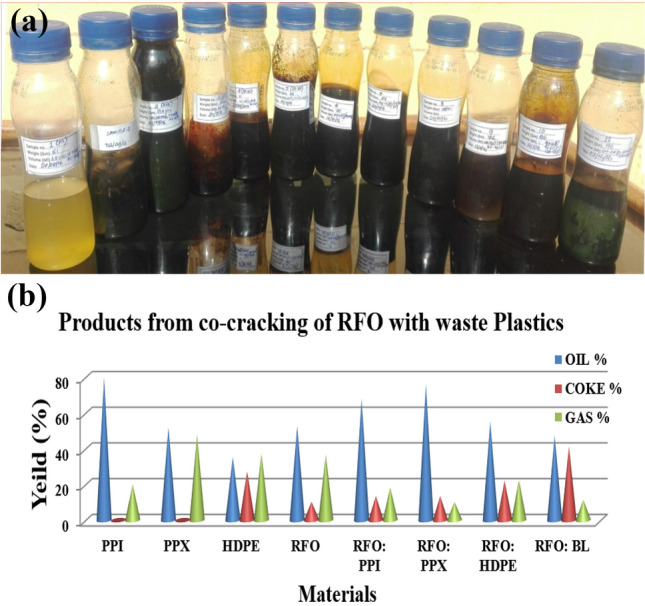
(**a**) Liquid product obtained from the co-cracking of RFO and waste plastics. (**b**) Graphical representation of products yield distribution from the binary co-cracking of RFO with plastics.

In a typical experiment, a self fabricated batch reactor was used to carry out the co-pyrolysis. Nitrogen gas was used for flushing the reactor and heated to the desired temperature of 773.15 K, at which most of the samples were decomposed completely with varying coke remaining in the reactor at the end of the cracking process. The samples (PPI, PPX, BL, HDPE, and RFO) and their combination in binary of 0.025 Kg each (1:1 wt/wt) were taken in a small container and loaded into the reactor after the reactor had attained the set temperature of 773.15 K, it was kept at this temperature as long as the liquid was produced from the condenser which varies from 1 to 3 h and resulting liquid product was used for the analysis in this experiement. The detail of the process and reactor used in the experiment has been reported elsewhere in our earlier paper^1^. The liquid products resulting from the co-cracking process^1^ were collected in a liquid–gas separator and maintained at room temperature. As such, the liquid was used as the sample for the analysis and characterization. The condensed liquid product resulting from the cracking process with different densities and colors, along with the product distributions, are shown in Fig. 1a,b.

The chemistry behind the pyrolysis process is that the feedstocks are subjected to heating in the absence of oxygen. As a result, the organic compounds break downs generating liquid and gaseous products. On the other hand, the inorganic compounds of the samples, free from organic matters, virtually remain unaffected in the solid fraction and, therefore, can be recovered for further utilization^23^. The process mechanism entails the formation of a carbenium ion through isomerization, random chain scission and β cleavage, hydrogen transfer, alkylation/oligomerization, and aromatization, which is influenced by the density, strength, and distribution of the acid sites of the catalyst. These reactions determine the products resulting from the process. A solid catalyst like zeolites mostly favors hydrogen transfer reactions since it has many acid sites^24–26^.

### Liquid product analyses

#### FT-IR studies

The FT-IR, make NICOLET of USA; model IMPACT 410 with 4cm^-1^resolutions was employed to determine the functional groups in the product. The IR spectra were recorded as a thin film between KBr windows. A total of forty scans were provided for a better signal and noise ratio.

#### NMR studies

^1^H and ^13^C-NMR spectra were determined using 600 MHz Nuclear Magnetic Resonance (NMR.) Spectrometer, Make Bruker, Model: ASCEND600. The 600 MHz FT NMR operates using Nuclear magnetic resonance spectroscopy as a working principle. It contains a room temperature smart probe with auto-tuning, ^1^H (Outer coil), and ^11^B, ^13^C, ^15^N, ^17^O, ^19^F, ^29^Si, ^31^P(Inner Coil) with a TCI Cryo probe. The liquid samples were diluted with CDCL_3_ containing 0.1 M chromium acetylacetone as a relaxation agent and Tetra Methyl Silane (TMS) as the internal reference.

#### GPC studies

Gel Permeation Chromatography (GPC) analysis was carried out using Chloroform as the solvent.SIL-20A autosampler, Shim-pack XR Series columns with its micro-plunger design, and the LC-20AD with the solvent-delivery system. During the operation, the oven temperature of 313.15 K and pressure of 72 kg/cm^2^ were maintained. The Pump Flow of 1 ml/min was maintained during the analysis.

#### Bomb calorimeter

Bomb calorimeter with 1108P oxygen bomb and 6775 digital thermometers, make Parr Instrument Co. U.S.A., model: Catalogue No. 13341EE, with working principle Isoperibol, time-controlled and dynamic system was used to analyze the calorific values of the samples.

#### Ultimate/CHNS (O) analyzer

While CHNS (O) Analyzer Make: ThermoFinnegan, Italy, Model series of FLASH EA 1112 was used in order to establish the percentages of Nitrogen (N), Hydrogen (H), Carbon (C), and Sulphur (S) based on the principle of "Dumas method" which oxidized the sample completely and instantaneous by "flash combustion”. The combustion products are separated by the chromatographic column and detected by the thermal conductivity detector (TCD), giving output signals proportionate to the concentration of the individual components present in the mixture.

#### Density

Pycnometer methods, using Pycnometer of Spherical pattern, 25 cm^3^ volume with accurate stopper, made from Borosilicate glass used in determining the thickness of the liquid product derived from co-cracking of RFO with various waste plastics (PPI, PPX, HDPE, BL) and their individuals. The experiment was carried out at a room temperature of 296.15 K. Distilled water with a density of 997.53 kg/m^3^. The density of the liquid was determined by employing the simple yet reliable methods of mass divided by volume was used.

#### Flashpoint and pour point

The Stanhope Seta PM-93, fully automated Pensky-Martens apparatus and standard glassware, combines strict method conformance with the latest control technology with an accurate and safe analysis machine. Standards of ASTM D93, IP 34, ISO 2719, DIN 51,758. Test methods of IS: 1448[P: 21]:2003 and IS: 1448[P: 10]:2003 were employed at 20–296.15 K environmental conditions and humidity at normal temperature, and pressure was maintained during the process.

## Results and discussion

### GPC analysis of the liquid products

The molecular weight distributions of the liquid products resulting from the co-cracking of RFOwith various plastics (PPI, PPX, HDPE, and BL) are shown in Table 1. The liquid product resulting from the cracking of RFO alone was analyzed using the Gel Permeation chromatography technique to establish the molecular weight distribution (MWD) of the liquid. It was observed that the liquids product showed the number average molecular weight (Mn), weight average molecular weight (Mw), and average molecular weight (Mz) was found to be 90, 206, and 409, respectively. The variance in the molecular weight distribution is reflected by the higher polydispersity, as shown in Table 1; in concurrence from the ideal compound as per Gaussian distribution, the broader range of the compounds present in the sample is indicated by the higher polydispersity (PD).

**Table 1:** Molecular weight distribution of liquid products obtained from the co-cracking of RFO and Plastics.

Sl. no	Sample	Molecular Wt (%)	Mn	Mw	Mz	Mz + 1	Polydispersity (Mw/Mn)
1	RFO	100	90	206	409	715	2.286
3	PPI	32.80	269	377	523	656	1.400
		57.30	65	68	72	76	1.058
		9.90	21	22	23	23	1.042
4	HDPE	35.78	544	648	764	883	1.192
		58.87	69	76	84	93	1.101
		5.36	21	22	23	23	1.041
5	BL	53.05	˂100	–	–	–	–
		46.95	172	201	–	–	1.168
7	RFO+ PPI	81.31	44	78	165	366	1.761
		18.69	10	11	11	12	1.076
8	RFO+ BL	68.38	54	89	175	359	1.646
		17.53	13	14	14	15	1.064
		7.82	5	6	6	6	1.048
		6.28	2	2	2	3	1.071
9	RFO+ HDPE	100	84	182	397	758	2.163

*Mn* number average molecular weight, *Mw* weight average molecular weight, *Mz* average molecular weight, *Mw/Mn* polydispersity, *Mz + 1* average molecular weight.

The majority of the liquid products (57.31 percent) have a weight average and number average molecular weight of 141 and 82, respectively, with a PD of 1.714, according to the examination of liquid products derived from the cracking of PPX. Mw of 25 and Mn of 24 with a PD of 1.037 was detected in about 19 percent of the liquid. However, the liquid product (83.91 percent) had Mw of 77 and Mn 48, respectively, with a PD of 1.617. The PD of RFO suggests that the generated liquid is very complex, meaning that liquid products contain a wider variety of complex compounds^24^. The "numerical average molecular weight" of liquids obtained from the binary co-cracking of PPX and RFO was similar to that of liquids derived from PPX. Furthermore, when RFO and PPX were co-processed jointly, an overall drop in the distributions of molecular weights of the liquid product was observed. Further, the co-cracked derived liquid showed a decline in the polydispersity index from that of the individual. The result clearly suggests that the putrefied products of polypropylene (PPX) in the process could further interact with the resulting pyrolysis product from RFO, thereby enabling a further-cracking reaction. Moreover, a decrease in the polydispersity index was observed in the liquid obtained from the co-cracking of the PPX and RFO in comparison with the theoretical average values of the individual samples, indicating a synergism as a result of the co-cracking reaction. The detailed GPC characterization of the liquid product from the co-cracking of RFO + PPX has been reported in another paper^27^.

From the liquid products acquired from pyrolysis of PPI alone, we have observed that 32.80% of the liquid products were found to have an Mn and Mw of 269 and 377, respectively. Around 57.30% of the liquid was found to have Mn and Mw of 65 and 68, respectively, with a polydispersity of 1.058. However, co-cracking of RFO and PPI yielded liquid product with 81% of liquid showing an Mn and Mw of 44 and 78, respectively, with a polydispersity of 1.761. The data demonstrates that the Mn and Mw of the liquid products from RFO and PPI co-cracking were reduced significantly from that of the individual. This implies that the decomposed products of PPI can interact with the products resulting from the cracking of RFO, thereby making the co-cracking reaction possible. Further, the polydispersity index was found to have decreased compared to the theoretical average value of the liquids derived from the individual RFO and PPI.

The liquid acquired from the cracking of HDPE showed a range of molecular weights. The maximum of the liquid products (58.87%) had an Mn and Mw of 69 and 76, respectively. Interestingly, the Mn and Mw of the liquid products resulting from the co-cracking of RFO and HDPE were 84 and 182, respectively, with a polydispersity of 2.16. Accordingly, it was observed that there was an overall reduction in the molecular weight distributions of the liquid obtained from the co-cracking of RFO and HDPE.

This implies a definite interface between the reacting species resulting from the cracking of HDPE and RFO, which produces a chemical species with lower molecular weight. The reduction in the molecular weight of the liquid products resulting from co-cracking of RFO and HDPE may be due to the decomposition of HDPE by a radical chain mechanism resulting in unsaturated and monomers or shorter chains molecules.

Consequently, the reactive radicals resulting from the decomposition of HDPE can play a role in the saturation of the residue fragments, ultimately decreasing the molecular weight of the liquid resulting from the co-cracking of RFO and HDPE.

The GPC analysis result of liquid products resulting from the pyrolysis of bakelite, showing its molecular weight distribution, has been shown in Table 1. Around 53.07% of the liquid products have been found to have a molecular weight below 100, while the remaining 46.95% of the liquids product were found to have 172 and 201 as the Mn and Mw, respectively. Therefore, it was observed that 53.05% of the liquid product obtained from the cracking of bakelite has low-viscosity. However, the liquid products resulting from the co-cracking of Residual fuel oil and bakelite, 7.82% of the liquids products showed a molecular weight distribution of 5(Mn) and 6(Mw). Around 68.38% of the liquid products have number average and weight average molecular weights of 54 and 89, respectively. The molecular weight distribution of the liquid products resulting from the co-cracking of RFO with BL^28^ was found to be lower than that of the individual cracking. This may be explained by the fact that the free radicals generated for the decomposition of RFO interact with the pyrolysis products of BL. The RFO may have therefore acted as a hydrogen donor solvent during the co-cracking process with BL, thereby facilitating better interaction between the two samples during the cracking process. Around 17% of liquid products showed Mn and Mw of 13 and 14, respectively. Therefore, the molecular weight distribution of the liquid products resulting from the co-cracking was found to be lower than when they were cracked individually. The observation clearly suggested that there was an interaction between reacting species during the co-cracking process of RFO and BL.

### FT-IR analysis of the liquid products

The FT-IR analysis is an effective technique used to distinguish different functional groups that may be present in the samples and products obtained from the co-cracking. During the analysis process, the infrared light upon interfacing with the pyrolysis oil, elongation, and contraction of the chemical bond occurs, and thereby at a specific wavelength range, absorption of the infrared radiation will occur, regardless of the variation in the molecular structure.

**Figure 2:**
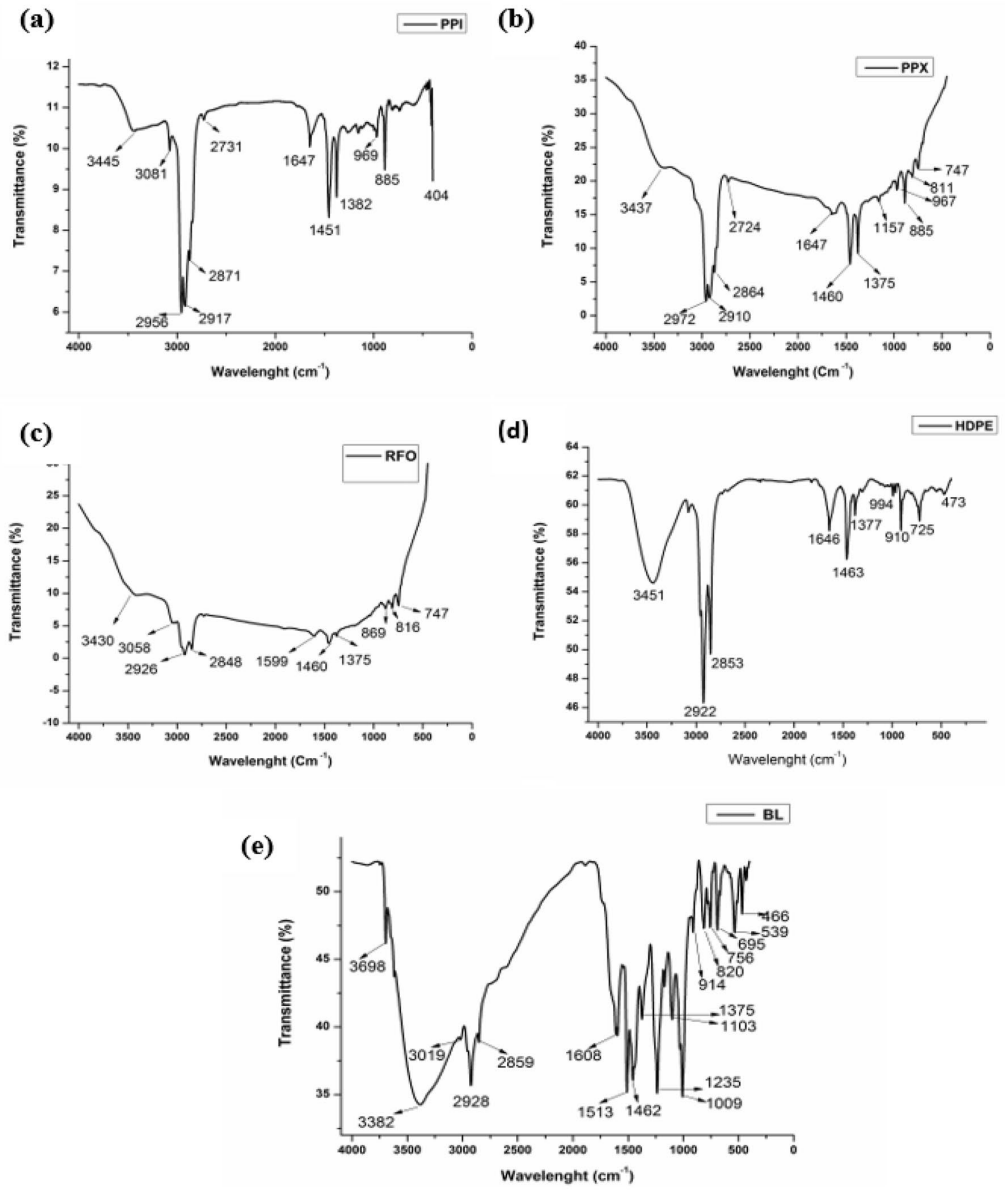
FT-IR spectra of the liquid product obtained from the co-cracking of (**a**) PPI (**b**) PPX (**c**) RFO (**d**) HDPE (**e**) BL.

Figure 2a,b shows the FT-IR spectra of PPI and PPX at 3449 and 3437 cm^−1^ is an indication of the presence of proton from alcohol. At the same time, the peak present in the 3081 cm^−1^ in the PPI spectra may be due to the presence of =C–H stretching vibration from the alkene functional group. The peak at 2956 cm^−1^ (PPI), and 2972 cm^−1^ (PPX) may be due to the presence of alkane, C–H stretching with saturated carbons, while the peak at 2917 (PPI) and 2910 cm^−1^ (PPX) may be due to the presence of C-H_2_ stretching of alkanes. The peak at 2871 cm^−1^ spectra of PPI may be an indication of the presence of aliphatic hydrogen (C-H stretching), similar result was also observed by Chun^29^. The FT-IR spectra of liquid obtained from the cracking of RFO given in Fig. 2c show the presence of the O–H functional group. The peak at 2926 cm^−1^ indicates the presence of CH_3_ groups, while the peak at 2848 cm^−1^ indicates the presence of aliphatic CH_3_, CH_2,_ and CH, but it is likely that CH_2_ is the most dominant group. However, it was not feasible to conclude whether the CH_2_ groups were part of the aliphatic chains in cycloparaffins or hydroaromatic structures. The band visible at 1599 cm^−1^ may be due to the presence of C=C bonding which may have also been intensified by the oxygen-containing functional groups^30^. The peak at 1460 cm^−1^ may be partly due to the CH_2_ groups, but contribution may have been sourced from the CH_3_ groups, aromatic C=C bonds, and OH group with a strong hydrogen bond.

**Figure 3:**
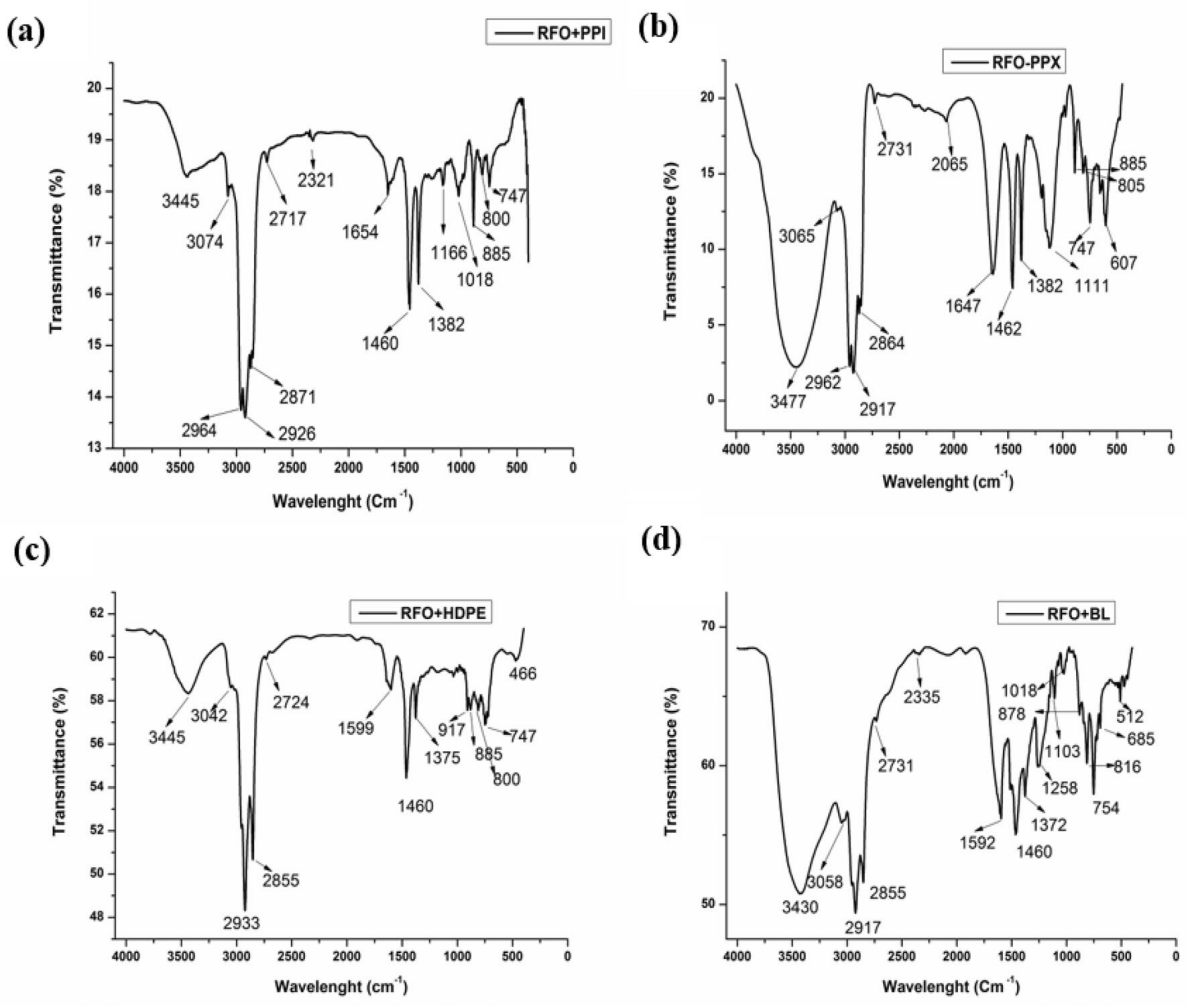
FT-IR spectra of the liquid product obtained from the co-cracking of (**a**) RFO + PPI (**b**) RFO + PPX (**c**) RFO + HDPE (**d**) RFO + BL.

Figure 2d shows the spectra of HDPE, which are primarily similar to that of the PPI and PPX. The peak at 3451 cm^−1^ indicates the presence of O–H groups, while the presence of the alkanes group is indicated by the peak at 2922 cm^−1^, while the peak at 2853 cm^−1^ may be an indication of the existence of aliphatic CH groups. The peak at 1646 cm^−1^ confirms the presence of alkene in the liquid since alkene peaks occur between 1640 and 1680 cm^−1^. Further, the peak at 994 cm^−1^ may indicate the existence of CH bending vibration, which suggests that there are alkene groups present in the products^31^. The peak at 725 cm^−1^ may indicate the presence of C–H cyclic deformation, thereby suggesting that either aromatic or, more probably –CH_2_ splits due to interaction in the long molecular setup. The liquid product derived from the cracking of BL showed a peak at 3382 cm^−1^ in the FT-IR spectra Fig. 2e, indicating the stretching vibrations of O–H, which occur between 3202–3430 cm^−1^, further implying the presence of alcohols and phenols^9^. The presence of hydrogen-bonded –OH groups was observed in the liquid product obtained from the cracking of RFO + BL with an abroad peak at 3430 cm^−1^ in the FT-IR spectra. The FT-IR spectra of RFO + PPI, as shown in Fig. 3a, resemble more to that of the spectra of the liquid product obtained from the cracking of PPI though the overall spectra were a mixture of the spectra from both the RFO and PPI. The weak peak at 1460 cm^−1^ in the FT-IR spectra of RFO became more intensified in the liquid obtained from the co-cracking of RFO + PPI, indicating the presence of alkanes by C–H scissoring and bending or simply a hydrocarbon with another peak at 1375 cm^−1^ for RFO but shifted to 1382 cm^−1^ in the co-cracked liquid of RFO + PPI. The FT-IR spectra of the liquid products obtained from the co-cracking of RFO, PPX, and RFO + PPX given in Fig. 2b has been reported elsewhere in other paper^27^. However, the FT-IR spectra of the liquid resulting from the co-cracking of RFO + HDPE given in Fig. 3c suggest the presence of both aromatic and aliphatic functional groups. The peaks between 675 to 900 cm^−1^ and a peak between 3000 and 3050 cm^−1^ show a presence of aromatic compounds in the products. The peak at around 1460 cm^−1^ present in all spectra of RFO, BL, and RFO + BL could be an indication of the presence of CH_2_ Methylene groups. The lists of the spectra of prominent peaks of the sample are reported in Table 2. The peak at 1030 cm^−1^ in Figs. 2c and 3c may be due to the presence of S=O in the RFO sample.

**Table 2:** List of the prominent peaks of FTIR analysis of the liquid products obtained from the co-cracking of RFO with waste plastics.

Sl no	Peaks	PPX	PPI	HDPE	BL	RFO	RFO + PPI	RFO + PPX	RFO + HDPE	RFO + BL	Bond	Functional Group
1	3698	x	x	x	✓	x	x	x	x	x	O–H stretch free	Phenol
2	3445	✓	✓	✓	x	x	✓	✓	✓	✓	O–H	Alcohol
3	3382	x	x	x	✓	x	x	x	x	x	O–H stretching, Intermolecular bonding	Alcohol
4	3081	✓	✓	x	x	✓	✓	✓	✓	✓	= C-H stretching vibration	Alkenes
5	3019	x	x	x	✓	✓	x	x	x	x	N–H stretching vibration	Primary amine group
6	2956–2972	✓	✓	x	x	x	✓	✓	x	x	C–H stretching of saturated carbons	Alkane
7	2917–2922	✓	✓	✓	✓	✓	✓	✓	✓	✓	C–H_2_ stretching vibrations	Alkanes
8	2853–2855	✓	✓	✓	✓	✓	✓	✓	✓	✓	aliphatic CH groups	CH_2_
9	1647	✓	✓	✓	x	x	✓	✓	x	x	C = C stretching vibrations or OH of water	Alkenes
10	1460	✓	x	✓	✓	✓	✓	✓	✓	✓	aromatic C = C bonds or OH group, but it may be CH_2_ Deformation/CH_2_ in the case of HDPE	Methylene
11	1451	x	✓	x	x	x	x	x	x	x	C–H scissoring and bending vibrations	Alkanes
12	1375	✓	✓	✓	✓	✓	x	x	✓	✓	CH_3 _symmetric bending	Methyl groups
13	1382	x	x	x	x	x	✓	✓	x	x		
14	1235	x	x	x	✓	x	x	x	x	x	C-O stretch indicating the presence of acid	Acid
15	994	x	x	✓	x	x	x	x	x	x	C-H bending vibration	alkene
16	910–917	x	x	✓	✓	x	x	x	✓	x	C–O stretching vibrations	
17	725	x	x	✓	x	x	x	x	x	x	C–H bending vibrations at frequency	Methyl
18	473	x	x	✓	x	x	x	x	x	x		
19	466	x	x	x	✓	x	x	x	✓	x	O–H Vibration	Alcohol

While peaks at 1647 cm^−1^ were present in both the spectra of PPX Fig. 2b and RFO Fig. 2c were not intense; however, an intense peak could be observed in the spectra of the co-cracked liquid sample of RFO + PPX Fig. 3b which may be due to the presence of O–H bond of water present in the sample or C=C–H stretching vibrations of alkenes. The change in the intensity of the peak at 1647 cm^−1^ upon the co-cracking stand demonstrates a strong interaction between the two feedstock and hence the synergistic effect of co-cracking. Further, the peak at 466 cm^−1^ observed in Fig. 2e may be due to the O–H present in the sample.

Similarly, the peak at around 1375 cm^−1^ in the FT-IR spectra of both RFO and BL is indicative of the presence of CH_3_ methyl groups, which have characteristics of bending absorption approximately around this wavelength, which was considerably lower in the case of liquid from the RFO + BL as shown in Fig. 3d.

### ^1^H and ^13^C NMR spectra analysis

The chemical shifts definition of the proton and carbon NMR study of the hydrocarbons are given in Table 3, and the average structural parameters of the liquid products obtained from the co-cracking are RFO with plastics which include PPX, PPI, HDPE, and BL are given in Table 4. The branched, straight-chain, and cyclic alkanes are responsible for the resonance signals observed in the spectra. The integration of the ^1^H NMR aliphatic regions shows that the liquid product from the polypropylene (PPI) contains 86.89% aliphatic hydrogen. However, in the liquid product of PPI, the aliphatic hydrogen distribution was mostly H_α_ (13.11%), H_CH3_ (29.51%), and H_β+γ_ (44.26%), respectively, and almost a similar observation was also made in the case of liquid obtained from the cracking of PPX, the ^1^H-NMR and ^13^C-NMR spectra of PPX. The liquid products derived from the pyrolysis of residual fuel oil were found to contain 87.50% of aliphatic hydrogen and 11.32% of aromatic hydrogen. The distribution of aliphatic hydrogen was mainly H_α_ (20.19%), H_CH3_ (17.31%), and H_β+γ_ (50%). But, due to the possible overlap of the resonance signals of cyclic and acyclic methyl hydrogens, the information on quantification of the amount of these proton types which can be extracted from these spectra is limited.

**Table 3:** Definitions of ^1^H and ^13^C NMR chemical shifts for hydrocarbons.

Parameters	Chemical shift (δ)	Definition
CA	100–150	% Aromatic carbon
CS	10.0–50.0	% Saturated aliphatic carbons
CP_α_	14.1	% Carbon of terminal methyl group of alkyl chain α CH_3_-(CH_2_)n- (n ≥ 4)
CP_β_	22.7	% Carbon of CH_2_ group β to terminal methyl of alkyl chain with n ≥ 4
CPn	29.6–30.1	% Carbon of γ or higher of alkyl chain CH_3_-CH_2_-CH_2_-(CH_2_)n-CH2-CH_2_
CP_γ_	32	% Carbon of γ CH_2_ in CH_3_-(CH2)_γ_-CH_2_-CH_2_-(CH_2_)_n_
CP		CPα + CPβ + CPn + CPγ
CAI	129.2–132.5	% Bridgehead (internal) aromatic carbon
CAH	100–130	% Protonated carbon
HA	9.0–6.0	% Aromatic proton
H _α_	4.0–2.0	% CH_3_, CH_2,_ and CH proton α to an aromatic ring
H_β+γ_	2.0–1.0	% CH_2_ and CH protons of alkyl chains β or further to ring and CH_3_ protons β to ring
H_CH3_	1.0–0.5	% CH_3_ proton of alkyl chains γ or further from aromatic ring or CH_3_ of saturated compounds
HS	4.0–0.5	% Aliphatic proton
C/H =	Carbon to hydrogen ratio = [2CS + CA/CS + CA]	
ACL =	Average chain length = 2CP/CPα	
R_A_ =	No. of aromatic rings per average molecular = ( 1 + CAI/2)

**Table 4:** Average structural parameters (Derived from ^1^H and ^13^C NMR data) of liquid products obtained from co-cracking of RFO with various types of plastics.

Sl no	Parameters	Liquid from								
		RFO	PPX	PPI	HDPE	BL	RFO + PPI	RFO + PPX	RFO + HDPE	RFO + BL
1	CA	30.77	29.33	20.90	6.90	80.00	23.19	18.18	6.90	10.34
2	CS	69.23	70.67	79.10	93.10	20.00	76.81	81.82	93.10	89.65
3	CP_α_	7.41	1.89	1.89	5.56	7.69	1.89	1.85	7.41	1.92
4	CP_β_	5.56	1.89	1.89	5.56	7.69	1.89	1.85	5.56	1.92
5	CP_n_	22.22	5.66	3.77	44.44	10.77	9.43	11.11	44.44	15.38
6	CP_γ_	1.85	1.89	1.89	3.70	7.69	1.89	1.85	3.70	1.92
7	CP	37.04	11.32	9.43	59.26	33.85	15.09	16.67	61.11	21.15
8	CAI	8.33	9.09	14.29	25	34.62	6.25	8.33	25.00	16.67
9	CAH	91.67	54.55	85.71	75.00	11.54	62.50	83.33	50.00	100
10	R_A_	5.16	5.54	8.14	13.50	18.31	4.12	5.16	13.5	9.33
11	ACL	10	12	10	21.33	8.80	16	18	16.50	22.00
12	H/C	1.69	1.71	1.79	1.93	1.20	1.77	1.82	1.93	1.90
13	HA	11.32	1.04	11.66	7.32	44.26	7.50	5.94	15.56	10
14	H_α_	20.19	5.75	13.11	7.04	32.65	3.48	5.05	12.99	11.67
15	H _β+γ_	50.00	33.33	44.26	73.24	10.20	42.61	53.54	57.14	43.33
16	H_CH3_	17.31	59.77	29.51	11.27	2.04	46.09	35.35	11.69	33.33
17	HS	87.50	98.85	86.89	91.55	44.90	92.17	93.94	81.82	88.33

The ^1^H NMR spectrum of liquid obtained from the cracking of both PPX and PPI showed a high aliphatic character and resonance peaks visible in the spectra are due to the straight, branch, and cyclic chain aliphatic compounds. In the spectra obtained from the cracking of PPX, the aliphatic hydrogen distributions mainly consisted of H_β+γ_ (33.33%) and H_CH3_ (59.77%), and some H_α_ (5.75%). The ^13^C NMR Spectra of PPX liquid supported the observation made in the ^1^H NMR spectra showing a highly aliphatic character. The peaks at 44.44, 22.22, 29.76, and 30.31 ppm correspond to C_1_, C_2_, C_3,_ and C_4,_ respectively, of aliphatic long-chain compounds^32^. The relative presence of resonance signals in the 22.8–279 ppm and 32–40.72 ppm ranges are suggestive of the presence of significant amounts of branch aliphatic compounds. The quaternary substituted carbon in bridge carbon of fused ring system and aliphatic compounds normally have resonance spectra present higher than 40 ppm^32,33^. And therefore, numerous resonance peaks that have been recorded above 40 ppm may be related to quaternary substituted carbon in aliphatic compounds (since the pyrolytic liquid yield of polypropylene does not yield any cycloalkane or aromatic compounds)^34^. Examination of the integrated lines in the region above 40 ppm, it was found that approximately 46.05% of the total carbon atoms are present as quaternary carbons.

In the ^1^H-NMR and ^13^C-NMR spectra of the liquid product resulting from the cracking of RFO, it was found that the carbon types acquired from the cracking of RFO were disseminated between aromatic (100–150 ppm) and aliphatic (10–50 ppm) structures but distinctive sections can be identified within these bands for specific structures. The result, classification, and types of carbon distributions are given in Table 4. The resonance signal of carbon atom belonging to the long straight paraffinic chain (14.1, 22.7, 28.9, 29.1, 29.3, 29.5, 29.7, 31.9, and 33.8) was prominent, and the peak with the highest relative intense among them was observed at 29.7 ppm. Further, the relatively weak intensity at 19.7 ppm in contrast to that at 14.1 ppm implies that the extent of the branching is low.

^1^H NMR spectrum of the liquid product obtained from co-cracking of RFO + PPX mainly constituted by aliphatic components of up to 93.94% for PPX + RFO. For the co-cracking PPX + RFO, the distribution of aliphatic hydrogen was mostly H_β+γ_ (53.54%), H_CH3_ (35.35%), and some H_α_ (5.05%). The co-cracking has increased the H/C ratio.

The ^1^H-NMR and ^13^C-NMR spectrum of RFO + PPI exhibits that aliphatic components represent the predominant molecular species present (92.17%). The distributions of aliphatic hydrogen were mainly H_β+γ_ (42.61%) and H_CH3_ (46.093%), and some H_α_ (3.48). Olefinic hydrogens were found to be 3.48%. The aromatic species constitutes about 7.50% of the total hydrogens present in the liquids products. Further, it was observed that there was a decrease in the percentages of methyl hydrogen (H_CH3_) of the liquids resulting from the co-cracking of RFO + PPI in comparison with their theoretical averages from RFO and PPI individually. From the ^13^C-NMR spectrum, it was observed that the liquid products resulting from the co-cracking of RFO + PPI have a quite complex aliphatic region.

It was observed that the percentages of cyclic or branched compounds increased relative to their theoretical averages. From the ^1^H-NMR and ^13^C-NMR spectra of liquid products resulting from the cracking of HDPE, it was found that it contained 7.32% aromatic proton and 91.55% aliphatic hydrogen. Aliphatic hydrogens were mainly H_β+γ_ (73.24%) and H_α_ (7.04%), and some H_CH3_ (11.27%); these aliphatic groups could be present as alkyl groups attached to an aromatic ring. The ^13^C NMR spectrum further reveals that the liquid products from the cracking of HDPE showed no peak at around 67.9 ppm that signifying the absence of acetylenic compounds.

The spectra show that the liquid has 75% protonated aromatic carbon, and 93.10% of saturated aliphatic carbons were found in the liquid product. However, the liquid product acquired from the co-cracking of HDPE with RFOwas found to have 15.56% of the aromatic proton, which is higher than that of the HDPE alone, but lower aliphatic hydrogen with 81.82%. The aliphatic hydrogen consistsof Hα (12.99%), H_β+γ_ (57.14%), and H_CH3_ (11.69%). The content of protonated aromatic carbons was found to have decreased to 50% for the liquid product resulting from the co-cracking of RFO + HDPE. However, the range of saturated aliphatic carbon increased to 93.10% due to co-cracking.

The^1^H-NMR spectra of the liquid product from BL showed approximately 2.04% of methyl hydrogens (C_CH3_). The presence of phenolic OH was indicated by a peak at 4.8 ppm downfield from TMS in the FT-IR spectra. The Liquid product contains 44.90% of aliphatic protons, which are constituted by the H_α_ (32.65%), H_β+γ_ (10.20%), and H_CH3_ (2.04%). Therefore, the percentage of CH_3_, CH_2,_ and CHα hydrogen attached to an aromatic ring is 32.65% of the total hydrogens. The ^13^C-NMR spectrum reveals that the liquid product from the BL contains approximately 20% of the total carbon in the liquid product that comes from aliphatic species. The band in the range of 120–130 ppm, which is very complex, may be attributed to the substituted aromatic and polycyclic aromatic species. The sharp peaks at 115.28, 124.32, and 129.52 ppm could be allotted to C-2, C-6, and C-3, C-5 of phenolic moiety's carbon atoms. However, the liquid products obtained from the co-cracking of RFO, BL, contain 88.33% aliphatic. The aliphatic protons distributions wereH_α_ (11.67%) and H_β+γ_(43.33%), and H_CH3_ (33.33%). The aromatic carbon was found to be 10.23%.

Therefore, the liquid product obtained from the co-cracking of RFO + BL has an aliphatic compound as the primary constituent of molecular species present, clearly demonstrated by the ^13^C-NMR spectrum. The liquid obtained from the co-cracking of RFO + BL was more similar to the one derived from the cracking of RFO. From the integrated data reported in Table 4, approximately 89.65% are made up of aliphatic hydrocarbons, and the remaining 10.34% of carbons are aromatic in nature. Moreover, it was found that the content of aliphatic carbon increases when RFO and BL were co-cracked together as compared with their individuals. The H/C ratio of the liquid products was found to have increased as a result of co-cracking.

### Ultimate analysis of the liquid products

Table 5 shows the percentage of elemental carbon in the feedstocks. The higher percentage of elemental carbon of the feedstocks indicates its suitability for the cracking process. The presence of nitrogen can be due to the additives found in the polymers. On the other hand, the occurrence of nitrogen can be related to the origin of crude oil and additives used in petroleum products. Consequently, nitrogen is found in the liquid products obtained from the co-cracking process. In all the samples, no sulfur content was found and related to the insignificant portion present in the solution. The properties of RFO are altered because of the source of crude oil and blending/additives added during the processing of fuel oil in the refinery. It was observed that the carbon element followed by hydrogen is the primary constituent of the liquid product obtained from the catalytic cracking of RFO with various waste plastics, including PPI, PPX, HDPE, and BL. A comparable observation was also made for the co-cracking liquid product from the binary combinations.

**Table 5:** Ultimate analysis of liquid product obtained from co-cracking of RFO with various plastics waste in the presence of ZSM-5 catalyst.

SL No	Materials	C	H	N	O
1	RFO	87.620	8.781	2.074	4.000
2	PPX	85.940	9.539	1.929	0.000
3	BL	72.944	7.161	4.696	13.646
4	PPI	88.021	10.965	1.014	0.000
5	HDPE	85.113	10.530	1.046	3.311
6	RFO + PPI	84.235	11.278	1.007	3.480
7	RFO + PPX	86.830	10.250	1.670	1.250
8	RFO + BL	81.820	9.600	3.212	0.000
9	RFO + HDPE	87.691	11.218	2.159	0.000

**Figure 4:**
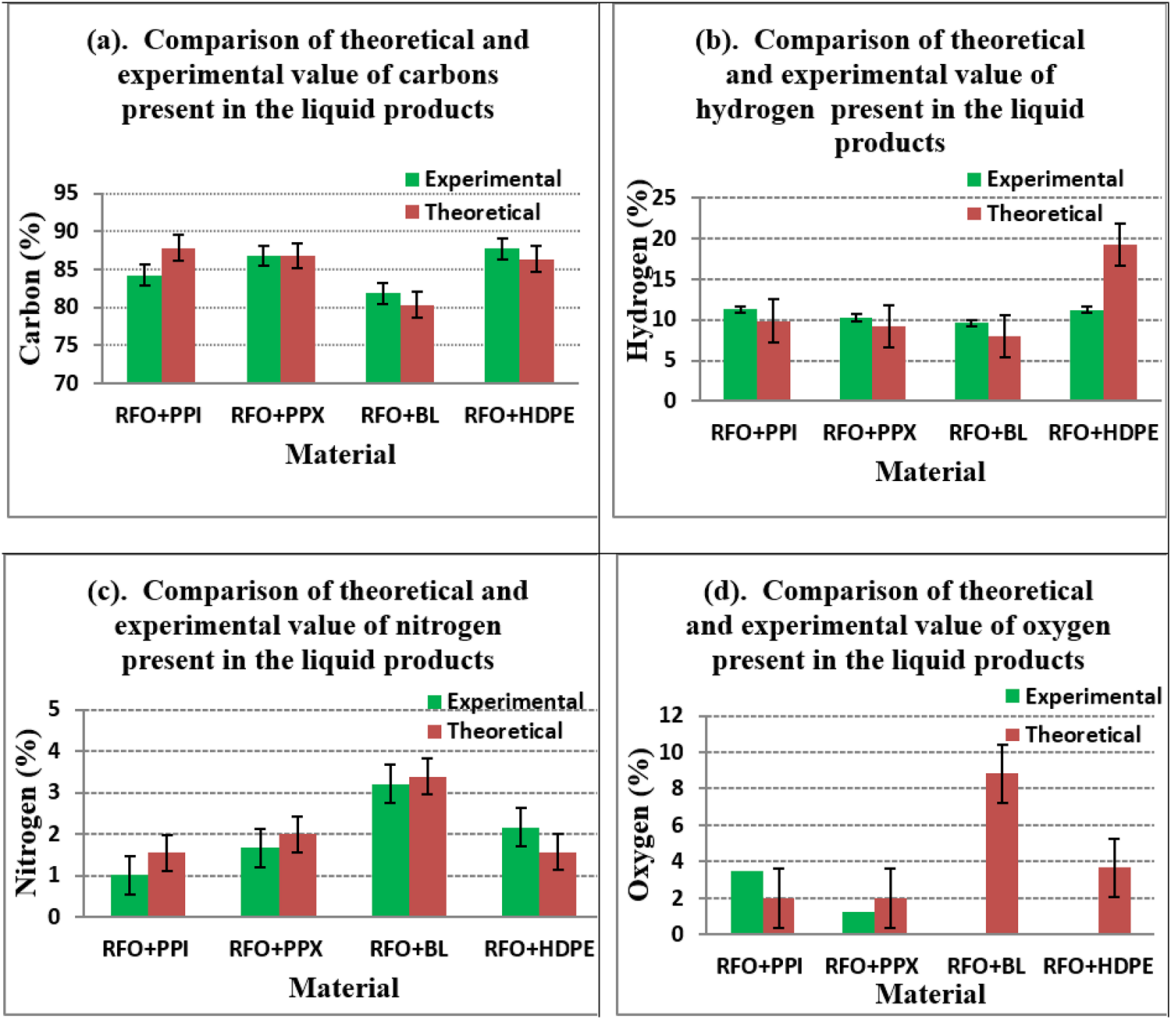
Comparison of theoretical and experimental values (**a**) Carbon (**b**) Hydrogen (**c**) Nitrogen (**d**) Oxygen.

The variation observed in the percentage of elements in the liquid products evidently implies the effect of co-cracking impacting the outcome of the process and hence its degree of characteristics. Figure 4a shows the comparison of the theoretical and experimental value of carbon content (%) in the liquid samples obtained from the co-cracking of RFO with various waste plastics in various combination mixtures.The theoretical values were determined by considering the experimental values (%) of the individual sample, and therefore the theoretical value of the binary sample will be the addition of the individual experimental values (%) of the two combining samples divided by 2. It was very clear from the figure that the experimental values of carbon content in the liquid products were higher than that of the theoretical value indicating a synergistic effect of co-cracking.

Hydrogen content constitutes the second major component of the molecule, as shown in Table 5. From the comparison given in Fig. 4b, the experimental values of hydrogen content were higher than that of the theoretical values. Figure 4c shows the comparison between the theoretical and experimental content (%) of nitrogen in the liquid product obtained from the co-cracking of RFO with the waste plastics, which includes PPX, PPI, BL, and HDPE.

The figure clearly shows that the nitrogen content in the liquid product was reduced by co-cracking except in the blending combination of RFO + HDPE. Figure 4d shows the comparison of the theoretical and experimental value of oxygen content in the liquid product derived from the co-cracking of RFO with the waste plastics. The overall results revealed that Nitrogen content was highest in the liquid product from BL, followed by the RFO, while the highest carbon content in the liquid product was recorded for PPI with 88.021% and lowest with the BL with 72.94% of carbon content in the liquid. The highest content of oxygen was recorded for the BL with 13.646%.

### Bomb calorimetry analysis of the liquid products

**Figure 5:**
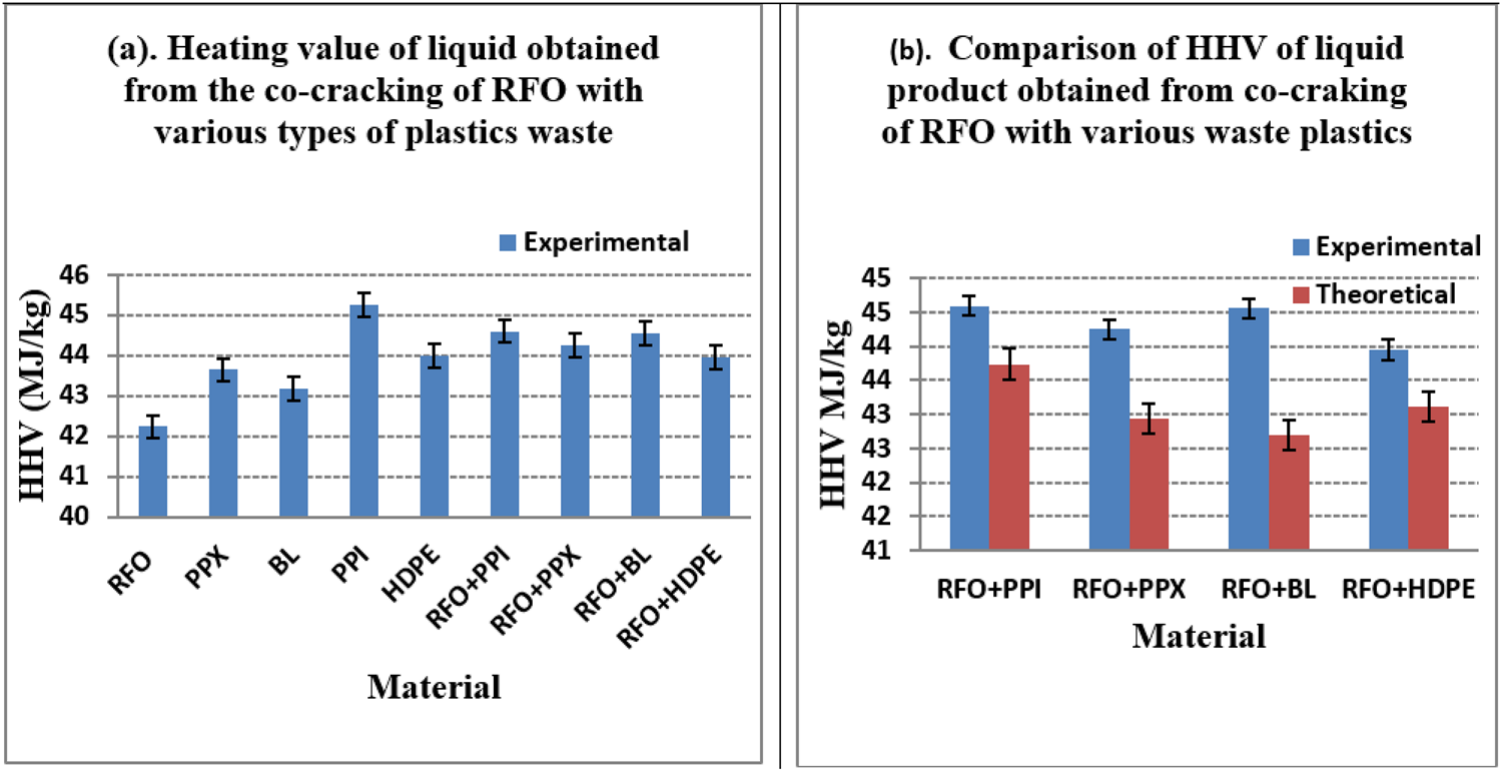
(**a**) Graphical representation of HHV values for the liquid obtained from the co-cracking of RFO with waste plastics, (**b**) Graphical representation of a comparison of theoretical and experimental HHV.

The calorific value of high heating values (HHV), which defines the energy released when a unit mass of fuel is burned in the presence of sufficient air, is one of the critical properties of fuel that indicates the efficiency of the fuel. The calorific values of the liquid product obtained from the co-cracking of RFO and various plastics waste and their individuals in the presence of ZSM-5 catalyst are given in Table 6. The calorific values of liquid products obtained, including those from the co-cracking of RFO and plastics (PPX, PPI, HDPE, and BL) in the binary combination of blending, were observed to have calorific values in the range of 42–45 M.J/kg. In Fig. 5a, the highest heating value was observed for the liquid products derived from the catalytic cracking of PPI with 45.24 MJ/kg. It was observed that the expected average theoretical value of calorific values of liquid products derived from the co-cracking of RFO with the waste plastics was lower than that of the experimental values as given in Table 6 and represented in Fig. 5b.

**Table 6:** Heating value of liquid obtained from the co-cracking of RFO with various types of plastics waste in the presence of ZSM-5 catalyst.

Sl no	Material	Experimental value (MJ/kg)	Theoretical value (MJ/kg)
1	RFO	42.23	**–**
2	PPX	43.64	**–**
3	BL	43.17	**–**
4	PPI	45.24	**–**
5	HDPE	43.99	**–**
6	RFO + PPI	44.60	43.74
7	RFO + PPX	44.25	42.93
8	RFO + BL	44.56	42.70
9	RFO + HDPE	43.95	43.11

All the HHV values obtained from the catalytic cracking of RFO and waste plastics are significantly high and are very close to the calorific values of diesel which has 44.94 MJ/kg^35^. While the HHV of PPI was even higher than that of the diesel, implying that liquid products obtained from cracking of the waste plastics and their co-cracking with RFO have a heating value equivalent to the conventional diesel and therefore have every prospect to be used as a source of transportation fuel like diesel by enhancing other properties.

### Density of the liquid products

Density is an important characteristic of any fuel oil. The economy of fuel consumption is directly related to the density of the fuel. Higher density fuels will have lower consumption. However, the oil with low density will consume more fuel which in turn will also impair the engine, implying that too high or too low density is not desirable in the fuel oil. The density of the fuel will also depend on the source, nature, and condition of the operation under which the samples are subjected, besides the characteristics of the co-cracking material^36^. The density of the liquid product derived from the co-cracking of RFO with various waste plastics is given in Table 7. It was observed that RFO had shown a density of 996 kg/m^3^ while PPX and PPI were witnessed to have a density of 778 and 777 kg/m^3^, respectively. In contrast, the liquid product derived from the co-cracking of RFOwith BL was found to have high viscosity and, therefore, could not determine the density using the pycnometer. On co-cracking of the RFO with various plastics, the density was found to have slightly increased (from 832–879 kg/m^3^) as compared with the density of the individuals; a similar observation was also found by Kumar and Singh^37^. Further, it was observed that while the co-cracking has reduced the density of the liquid product, the co-cracking of RFO with PPI and PPX has helped reduce the density more than that by co-cracking with HDPE and BL.

**Table 7:** Density of the liquid product obtained from the co-cracking of RFO with waste plastics in the presence of ZSM-5 catalyst.

Sl. no	Material	Density (kg/m^3^)
1	RFO	996
2	PPX	778
3	PPI	777
4	HDPE	800
5	BL	–
6	RFO + PPI	832
7	RFO + PPX	841
8	RFO + BL	103.8
9	RFO + HDPE	861

The density of the liquid product obtained from the co-cracking was found to be close to that of the diesel, kerosene, and both viscosity and density can be reduced by distillation^38^. The co-cracking liquid obtained from the process can be separated into different categories of diesel, gasoline, and kerosene according to their boiling temperature through fractional distillation^39,40^. Therefore, the result clearly suggests that conventional fuels like diesel oil and gas oil may be replaced by pyrolysis oil from the co-cracking of RFO with various plastics.

### Flash point and pour point of the liquid products

The flashpoint of liquid products is an important characteristic; knowing the flashpoint can help avoid fire hazards of the fuels during its transportation, handling, and use. The flashpoint of the liquid obtained from the catalytic cracking of RFO was found to be 302.45 K, as shown in Table 8. The flash point of individual plastics, including PPI, PPX, HDPE, and BL, was found to be lower than the room temperature, while the flashpoint of the liquid product derived from co-cracking of RFO + PPX was found to be 297.15 K. Other researchers have reported that the flashpoint for the waste plastics pyrolysis oil was found to be about 288.15 K which is lower than that of furnace oil, kerosene, and diesel and therefore indicates that the liquid oil can be easily handled^41^. The liquid product derived from the co-cracking waste lubricant oil with polyolefin (PE + PP + WMO) showed a lower flash point than that of the commercial diesel (> 328.15 K)^42^, while the flashpoint of HDPE was 283.15 K^37^. Therefore, the flashpoint from the co-cracking of RFO with waste plastics (PPI, PPX, HDPE, and BL) was lower than that of the commercial diesel, and therefore transportation and storing of the liquid product acquired through co-cracking of waste plastics with RFO may not be as safe as the diesel. However, the flashpoint may increase after fractional distillation of the liquid oil into its fraction. The flashpoint can also be increased by removing the lighter components such as gasoline and naphtha^41^.

**Table 8:** Flash Point and Pour Point of the liquid product derived from the co-cracking of RFO with various types of waste plastics (PPI, PPX, HDPE, and BL).

Sl no	Material	Flash point (K)	Pour point(K)
1	RFO	302.45	297.15
2	PPI	Below room temperature	Below − 308.15
3	PPX	Below room temperature	Below − 308.15
4	HDPE	Below room temperature	Below − 308.15
5	BL	Below room temperature	Below − 308.15
6	RFO + PPX	297.15	294.15
7	RFO + PPI	Below room temperature	291.15
8	RFO + HDPE	Below room temperature	306.15
9	RFO + BL	Below room temperature	292.15

Further, the pour point, which indicates the temperature at which the liquid oil will cease to flow upon cooling in a standard apparatus at a standard cooling rate, is a significant property that can help understand the suitability of the oil for use at low temperatures. The pour point of a liquid product derived from the co-cracking of RFO with waste plastics, including PPX, PPI, HDPE, and BL, is given in Table8. The pour point of liquid product from the cracking of RFO individuals was observed to be 297.15 K, while that of PPI, PPX, HDPE, and BL was found to be below 308.15 K. From Table 8, it can be concluded that, though the pour point may have decreased on co-cracking higher pour point was recorded with the liquid product derived from the co-cracking in the presence of HDPE. The high pour point of the liquid products clearly indicates that even if the liquid products fulfill the criteria of necessary properties of the fuel, they cannot be used in cold temperature areas^41^. The pour point of conventional diesel is in the range of − 313.15 to − 274.15 K^43^, pour point of HDPE was found to be 291.15 K^37^. However, some other study has reported that the pour point of pyrolytic liquid from HDPE was − 288.15 K^44^.

The liquid product derived from the co-cracking of RFO + PPI (291.15 K), and RFO + BL (192.15 K), is an acceptable pour point temperature in most geographic regions^37^. The variation in the pour point of the liquid product obtained from a similar material may be attributed to the use of the additives in the processing by the processing industries and the impurities present in the materials. Further, the source and nature of the samples also affect the characteristics of the resulting liquid product, including the pour point^36^.

## Conclusion

The weight distribution of molecules suggested that the products from the pyrolytic degradation of individual plastics can react with RFO during the co-cracking process resulting in the reduction of molecular weight as well as the complexity of the compound. The FT-IR spectrum of the liquid products from co-cracking was essentially found to have a greater presence of alkanes and alkenes compounds, with the primary elemental constituent of the liquid products resulting from the co-cracking, were found to be carbon, followed by hydrogen. An increase in the content of saturated aliphatic carbon and a decrease in the protonated aromatic carbons with aliphatic compounds as the primary constituent were observed from the spectra. However, the pattern of the spectra suggests that the aliphatic region was quite complex, with a significant amount of repeating structures. Experimentally obtained carbon element, as well as the calorific values, was higher than the theoretical average values expected on co-cracking, implying a synergistic effect of blending reaction. The calorific values of liquid products were observed to have calorific values in the range of 42–45 MJ/kg, which is equivalent to the conventional diesel. Further, the density of RFO was found to be 996 kg/m^3^, while PPX and PPI were observed to have a density of 778 and 777 kg/m^3^, respectively. However, the density of the liquid product obtained from the co-cracking was found to be close to that of the diesel and kerosene. The flashpoint of individual plastics was found to be lower than the room temperature, while the flashpoint of a liquid product derived from the co-cracking of RFO + PPX was found to be 297.15 K. The flashpoint from the co-cracking of RFO with waste plastics (PPI, PPX, HDPE, and BL) was lower than that of the commercial diesel. The pour point of the liquid product obtained from the co-cracking of RFO with various plastics used in the experiment has shown a pour point of approximately around 291.15–292.15 K, which is an acceptable pour point temperature in most the geographical regions. The co-cracking of RFO with waste plastics exhibits a positive synergism of the process; further, the liquid oil characteristics stand out to be a prospective source of energy and therefore have every prospect to be used as a source of transportation fuel like diesel by enhancing other properties.

## Data Availability

All data generated or analyzed during this study are included in this published article.
